# Effect of 13-valent pneumococcal conjugate vaccine on experimental carriage of *Streptococcus pneumoniae* serotype 6B in Blantyre, Malawi: a randomised controlled trial and controlled human infection study

**DOI:** 10.1016/S2666-5247(23)00178-7

**Published:** 2023-09

**Authors:** Dingase Dula, Ben Morton, Tarsizio Chikaonda, Anthony E Chirwa, Edna Nsomba, Vitumbiko Nkhoma, Clara Ngoliwa, Simon Sichone, Bridgette Galafa, Godwin Tembo, Mphatso Chaponda, Neema Toto, Raphael Kamng'ona, Lumbani Makhaza, Alfred Muyaya, Faith Thole, Evaristar Kudowa, Ashleigh Howard, Tinashe Kenny-Nyazika, John Ndaferankhande, Christopher Mkandawire, Gift Chiwala, Lorensio Chimgoneko, Ndaziona P K Banda, Jamie Rylance, Daniela Ferreira, Kondwani Jambo, Marc Y R Henrion, Stephen B Gordon, Malawi Accelerated Research in Vaccines, Experimental and Laboratory Systems (MARVELS) consortium

**Affiliations:** a Malawi Liverpool Wellcome Research Programme, Blantyre, Malawi; b Department of Clinical Sciences, Liverpool School of Tropical Medicine, Liverpool, UK; c Critical Care Department, Liverpool University Hospitals NHS Foundation Trust, Liverpool, UK; d Department of Medicine, Queen Elizabeth Central Hospital, Blantyre, Malawi; e School of Medicine, Kamuzu University of Health Sciences, Blantyre, Malawi; f Department of Paediatrics, University of Oxford, Oxford, UK

## Abstract

**Background:**

The effect of childhood pneumococcal conjugate vaccine implementation in Malawi is threatened by absence of herd effect. There is persistent vaccine-type pneumococcal carriage in both vaccinated children and the wider community. We aimed to use a human infection study to measure 13-valent pneumococcal conjugate vaccine (PCV13) efficacy against pneumococcal carriage.

**Methods:**

We did a double-blind, parallel-arm, randomised controlled trial investigating the efficacy of PCV13 or placebo against experimental pneumococcal carriage of *Streptococcus pneumoniae* serotype 6B (strain BHN418) among healthy adults (aged 18–40 years) from Blantyre, Malawi. We randomly assigned participants (1:1) to receive PCV13 or placebo. PCV13 and placebo doses were prepared by an unmasked pharmacist to maintain research team and participant masking with identification only by a randomisation identification number and barcode. 4 weeks after receiving either PCV13 or placebo, participants were challenged with 20 000 colony forming units (CFUs) per naris, 80 000 CFUs per naris, or 160 000 CFUs per naris by intranasal inoculation. The primary endpoint was experimental pneumococcal carriage, established by culture of nasal wash at 2, 7, and 14 days. Vaccine efficacy was estimated per protocol by means of a log-binomial model adjusting for inoculation dose. The trial is registered with the Pan African Clinical Trials Registry, PACTR202008503507113, and is now closed.

**Findings:**

Recruitment commenced on April 27, 2021 and the final visit was completed on Sept 12, 2022. 204 participants completed the study protocol (98 PCV13, 106 placebo). There were lower carriage rates in the vaccine group at all three inoculation doses (0 of 21 *vs* two [11%] of 19 at 20 000 CFUs per naris; six [18%] of 33 *vs* 12 [29%] of 41 at 80 000 CFUs per naris, and four [9%] of 44 *vs* 16 [35%] of 46 at 160 000 CFUs per naris). The overall carriage rate was lower in the vaccine group compared with the placebo group (ten [10%] of 98 *vs* 30 [28%] of 106; Fisher's p value=0·0013) and the vaccine efficacy against carriage was estimated at 62·4% (95% CI 27·7–80·4). There were no severe adverse events related to vaccination or inoculation of pneumococci.

**Interpretation:**

This is, to our knowledge, the first human challenge study to test the efficacy of a pneumococcal vaccine against pneumococcal carriage in Africa, which can now be used to establish vaccine-induced correlates of protection and compare alternative strategies to prevent pneumococcal carriage. This powerful tool could lead to new means to enhance reduction in pneumococcal carriage after vaccination.

**Funding:**

Wellcome Trust.

## Introduction

Pneumococcal conjugate vaccine has greatly reduced invasive pneumococcal disease worldwide, both by direct vaccine protection and by reduced carriage and transmission, leading to indirect protection (ie, the herd effect) for unvaccinated populations.^1^ In Malawi, the 13-valent pneumococcal conjugate vaccine (PCV13) was introduced in 2011 and has resulted in substantial reductions in invasive pneumococcal disease in vaccinated children.^2^ Unlike in the UK, however, there have been consistent observations of continued high rates of vaccine-type pneumococcal carriage among vaccinated children, and unvaccinated children and adults living with HIV in Malawi.^3^ This continued vaccine-type carriage, and the observed reduced herd effect in protection against disease,^4^ are a cause for concern.^5^

**Research in context**

**Evidence before this study**

We did an individual participant data meta-analysis (published May 4, 2023) to assess the safety of a pneumococcal controlled human infection model, searching for papers published between Jan 1, 2011, and June 30, 2022, with no language restrictions. By updating the search to April 1, 2023, we did not identify additional studies. We searched PubMed with the search terms “pneumococc*” OR “Streptococcus pneumoniae” AND “human infection model” OR “human infection stud*” OR “experimental human infection” OR “controlled human infection”. We found only one study designed to evaluate the effect of 13-valent pneumococcal conjugate vaccine (PCV13) vaccination on experimental pneumococcal colonisation. This double-blind, randomised controlled trial, done in Liverpool, UK, established that the risk ratio of experimental pneumococcal colonisation following PCV13 vaccination compared with control vaccine (hepatitis A) was 0·22 (95% CI 0·09–0·52; p<0·001). *Streptococcus pneumoniae* serotype 6B was used as the challenge agent, at a single dose of 80 000 colony forming units (CFUs) per 100 μL and natural pneumococcal carriage was observed in 6 of 100 participants in this study. Our group has previously shown feasibility of pneumococcal controlled human infection in Malawi with carriage rates of 44% at a dose of 80 000 CFUs per 100 μL (4 of 9 unvaccinated participants, 95% CI 13·7–78·8).

**Added value of this study**

We found that PCV13 was protective against experimentally induced pneumococcal carriage (serotype 6B) in healthy Malawian participants but to a lesser extent than observed in participants recruited to a similar UK-based trial. Episodes of natural pneumococcal carriage were high (42%) in our trial compared with published UK studies. We observed a greater PCV13 protective effect in participants without natural carriage (69·1%) compared with participants with natural carriage (51·7%) at the point of experimental pneumococcal inoculation. This is, we believe, the first vaccine trial that makes use of a bacterial human infection study in Africa. Our study provides important experimental data to inform epidemiological observations of high residual carriage of vaccine-serotype *S pneumoniae* after roll-out of PCV13 within the expanded programme on immunisation in sub-Saharan Africa.

**Implications of all the available evidence**

PCV13 vaccination was protective against experimental colonisation with pneumococcal serotype 6B in healthy adult volunteers from Malawi. An effective pneumococcal vaccine should interrupt population transmission through prevention of natural colonisation in vaccinated individuals. We have observed differences in protection compared with UK participants; this finding requires further evaluation by means of longitudinal immunological samples obtained from participants. Future research should pool data from the UK and Malawi populations to investigate correlates of protection. Our studies using pneumococcal controlled human infection could suggest means to improve schedules for current vaccines and to test prospective pneumococcal vaccines relevant to populations that need them most.

Vaccine efficacy is often reduced in low-resource settings compared with high-income countries. There are many reasons why this might be the case, including vaccine acceptability and uptake, and health systems issues such as cold chain integrity, high force of infection (high carriage rates in the community), pathogen diversity, and host immunity. In the case of the observed reduced herd effect following PCV13 implementation in infants in Malawi, it has been suggested that force of infection might explain the residual vaccine-type carriage in vaccinated children.^3^ There is high force of infection of both vaccine-type and non-vaccine type pneumococci in children and in susceptible populations such as people living with HIV or people living in crowded environments.^6^ It is possible, however, that host immunity developing in environments such as Malawi (these observations are true of many sub-Saharan African countries) results in a different mucosal defence to that observed in UK adults.^7^ In particular, the experience of seasonal viral infections, early life infections, and nutrition are very different between the two countries.^8^, ^9^, ^10^ Malawi has little seasonal variation of respiratory viruses, which are endemic at low levels all year round.^8^ Early life infections with bacterial^10^ and parasitic disease^11^ remain common and nutrition is variable with seasonal hunger.^12^ In this context, it is important to understand why vaccine-type pneumococcal carriage persists after vaccination, and to make every effort to optimise the efficacy of this important, expensive vaccine.

We previously developed a controlled human infection model (CHIM) of experimental human pneumococcal carriage (EHPC) in Liverpool, UK,^13^ to study mucosal immune determinants of pneumococcal carriage and transmission. The EHPC CHIM is safe, reproducible, and acceptable to volunteers in the UK and we used it to show PCV13 vaccine efficacy in prevention of experimental pneumococcal carriage in British university students.^14^ We then transferred the EHPC CHIM to Malawi, a low-resource high-transmission setting, and have previously shown feasibility and acceptability of this CHIM method for the first time in Africa.^15^, ^16^

In this study, we aimed to use the EHPC CHIM to establish the vaccine efficacy of PCV13 against experimental pneumococcal carriage in young healthy adults in Malawi.

## Methods

### Study design and participants

This was a double-blind, parallel-arm, randomised controlled trial investigating the efficacy of PCV13 or placebo (1:1) against experimental pneumococcal carriage of *Streptococcus pneumoniae* serotype 6B (strain BHN418; hereafter referred to as *S pneumoniae* 6B). The study was done according to our open access a priori published trial protocol (https://wellcomeopenresearch.org/articles/6-240/v2)^17^ with minor approved modifications reported here (see Statistical analysis section) and according to our prespecified statistical analysis plan.

The trial was approved in Malawi by the National Health Sciences Research Committee (16/07/2519) and Pharmacy Medicines and Regulatory Authority (PMRA/CTRC/III/10062020121) and in the UK by the Liverpool School of Tropical Medicine (20-021). Written informed consent was obtained for all participants.

Healthy adult participants were recruited from the community, workplaces, and colleges in Blantyre, Malawi. Study procedures took place in the Ward 3A research clinic, Queen Elizabeth Central Hospital (Blantyre, Malawi). Recruitment commenced on April 27, 2021 and the final visit was completed on Sept 12, 2022. A detailed description of screening, eligibility and recruitment procedures is described elsewhere.^17^ Briefly, prospective adult participants (aged 18–40 years) were screened by means of a combination of medical history, physical examination, and clinical laboratory tests to confirm eligibility before randomisation. Participants were excluded if at increased risk of invasive pneumococcal disease (eg, pregnancy or HIV infection), or they were close social contacts of individuals at increased risk (eg, breastfeeding an infant), or showed natural carriage of *S pneumoniae* 6B (precluding elucidation of primary outcome). Participants with natural carriage of pneumococcal serotypes other than 6B were not excluded. In an amendment from our published protocol, participants who tested positive for SARS-CoV-2 (all testing done by PCR) after inoculation were not automatically excluded from the trial but followed up to planned study exit. These participants were not included in the per-protocol analysis.

### Randomisation and masking

Per protocol,^17^ we powered the study to detect a 40% reduction in experimental colonisation from a baseline of 60% (control) to 36% (PCV13) in participants allocated to the 80 000 colony forming units (CFUs) per naris pneumococcal inoculation group (n=70 per group). Another 40 participants had been planned to be randomly assigned at 20 000 CFUs per naris and 20 participants at 160 000 CFUs per naris for an exploratory analysis of the dose (inoculation)–response (experimental carriage) curve. However, on the basis of lower than expected observed carriage rates in masked data (27% observed carriage *vs* 48% expected carriage across both study groups), the sample size was changed to 40 participants at 20 000 CFUs per naris dose, 80 at 80 000 CFUs per naris dose, and 80 at 160 000 CFUs per naris dose. The primary analysis was changed from the original plans (but a priori before data analysis commenced and prespecified in the statistical analysis plan) to a log-binomial model adjusting for inoculation dose, thereby allowing data from all inoculation doses to be used for the primary analysis.

After confirmation of eligibility, participants were randomly assigned (1:1) to receive intramuscular PCV13 (Prevenar-13 containing serotypes 1, 3, 4, 5, 6A, 6B, 7F, 9V, 14, 18C, 19A, 19F, and 23F; Pfizer, New York, NY, USA) or 0·9% normal saline (control). Research staff (clinical and laboratory) and participants were masked to vaccine allocation. The trial pharmacist (designated Qualified Person, JN) was unmasked to allocation and prepared intervention and control vaccines (identical in appearance and labelled A or B) on the day of randomisation. Three randomisation lists were generated by an independent statistician by means of code written by the trial statistician (MYRH) for each pneumococcal inoculation dose (20 000, 80 000, and 160 000 CFUs per naris) and prospectively uploaded to electronic case report forms with robust allocation procedures done per published protocol.^17^ The decision to test these doses was informed by previous work in pneumococcal CHIM development.^13^, ^15^ Randomisation to PCV13 or saline vaccination did not influence inoculation dose administered, with balanced allocation within each dosage group.^17^ The randomisation method used an allocation ratio of 1:1 between study groups and was a block randomisation procedure, which made use of variable block sizes from four to eight participants. The randomisation was implemented by means of the blockrand library for the R environment for statistical computation.^18^

### Procedures

28 days following vaccination, participants underwent nasal inoculation with *S pneumoniae* 6B to establish experimental human pneumococcal carriage with a method that we have used safely in Liverpool for more than 10 years.^19^ Briefly, experimental inocula of *S pneumoniae* 6B were prepared at the Liverpool School of Tropical Medicine, UK, following established standard operating procedures, including purity checks. Inocula were shipped to Malawi at −80°C and viability, bacterial count, and purity were confirmed from selected samples before human intranasal instillation. On each experimental day, a sample was thawed, centrifuged, and washed before resuspension in 0·9% normal saline at the prespecified concentration. The prepared inoculum was taken immediately (less than 5 min transit) to the clinical area where 100 μL was instilled into each nostril of each participant. Serial dilutions of the inocula were plated (pre-inoculation and post-inoculation) onto blood agar for dose confirmation. Owing to lower than expected total carriage rates observed in the 80 000 CFUs per naris group, a protocol modification was made (approved by the data, safety, and monitoring committee and research ethics committee) to escalate to 160 000 CFUs per naris after 81 participants had been inoculated at the 80 000 CFUs per naris dose. The first 42 participants were inoculated with 20 000 CFUs per 100 μL, the next 81 participants with 80 000 CFUs per 100 μL and the final 98 with 160 000 CFUs per 100 μL.

Nasal wash samples were collected immediately before and 28 days after vaccination, and after inoculation on days 2, 7, and 14. Sample collection and processing has been previously described.^15^, ^19^ Briefly, 5 mL of 0·9% saline was instilled into each naris, retained for 10 s and expelled in to a petri dish; this was repeated twice (10 mL per naris, 20 mL total). The recovered sample was immediately transferred to the laboratory on ice. On receipt in the laboratory, the sample was centrifuged at 3400 g for 10 min. Following centrifugation, 1 mL samples of the supernatant were stored at −80°C in ten pre-labelled tubes. The nasal wash bacterial pellet was resuspended in 100 μL of skim milk, tryptone, glucose, and glycerol with 20 μL plated onto Columbia sheep Blood Agar (Oxoid, Basingstoke, UK) containing 5 μg/mL gentamicin for pneumococcal isolation. Eight serial 1:10 dilutions were made from 20 μL of the suspension by means of the Miles and Misra method to establish the number of CFUs. All plates were incubated at 370°C with 5% CO_2_ for 18–24 h and inspected by two team members (ie, two of either TC, SS, BG, FT, or CM) to identify pneumococcal colonies by colony morphology. Colonies were then subcultured for optochin sensitivity, bile solubility, and serotyping. Pneumococcal serotypes were confirmed by latex agglutination by means of Immulex Pneumotest reagents (Statens Serum Institute, Copenhagen, Denmark). 800 μL skim milk, tryptone, glucose, and glycerol was added to the remaining nasal wash pellet and divided into three vials (300 μL into the first two tubes and the remainder into the third vial) and stored at −80°C for later molecular detection of pneumococci.

Genomic bacterial DNA was isolated from nasal wash pellets stored in skim milk, tryptone, glucose, and glycerol by means of the Agowa Mag mini-DNA extraction kit (LGC Genomics, Berlin, Germany) as per manufacturer's instruction by means of one of the three samples described previously. The extracted DNA was stored at −20°C for use in *lytA*/*cpsA* multiplex PCR. Sequences of the primers and probes used to do PCR were: *lytA* forward primer 5ʹ-ACGCAATCTAGCAGATGAAGCA-3ʹ; *lytA* reverse primer 5ʹ-TCGTGCGTTTTAATTCCAGCT-3ʹ; *lytA* probe 5ʹ-TGCCGAAAACGCTTGATACAGGGAG-3ʹʹ, and *cpsA* forward primer 5ʹ- AAGTTTGCACTAGAGTATGGGAAGGT-3ʹ; *cpsA* reverse primer 5ʹ-ACATTATGTCCATGTCTTCGATACAAG-3ʹ; and *cpsA* probe 5ʹ-TGTTCTGCCCTGAGCAACTGG-3ʹ. The reaction mixture of 20 μl contained 1 μL of each of the primers, 0·4 μL of each of the probes, 2·7 μL of PCR grade water, 10 μL of qScript XLT 1-Step RT-qPCR ToughMix (Quantabio, Cummings Centre, Beverly, MA, USA), and 2·5 μL of sample in a set of duplicate wells. The quantitative PCR (qPCR) reaction was run on a QuantStudio 7 Flex machine (Applied Biosystems, Lincoln Centre Drive, Foster City, CA, USA) with the following programme: 95°C for 10 min; followed by 40 cycles of 95°C for 15 s and then 60°C for 1 min.

### Outcomes

The primary endpoint was nasal carriage of *S pneumoniae* 6B established by microbiological culture of nasal wash at any time after inoculation (sampling timepoints days 2, 7, and 14 post-inoculation; participants expected to attend all visits). Nasal wash samples were recorded as positive if the *lytA*/*cpsA* PCR was positive at less than 40 cycles in duplicate samples. Adverse effects, and the effect of natural carriage at recruitment were included as secondary outcomes and are reported here.

### Statistical analysis

Data were collected electronically, with laboratory results pushed onto the database once they were available. Data collection tablets included range and consistency checks. Statistical analyses were done according to an a priori statistical analysis plan by means of R version 4.0.2 (R Development Core Team, Vienna, Austria) and GraphPad Prism version 9.0.0 (Graph-Pad Software, San Diego, CA, USA). Non-normally distributed quantitative measurements are summarised by the median (IQR). Exact binomial CIs are reported for estimated proportions. For the primary analysis, the binary response of experimental carriage was compared between study groups by means of a log-binomial model adjusting for inoculation dose group.

Our revised power calculation assumed the overall carriage proportions to be 5% (on the basis of observed, masked data at the time of protocol amendment) in the 20 000 CFUs per naris inoculation dose group, 27% in the 80 000 CFUs per naris dose group, and 48% in the 160 000 CFUs per naris dose group. We planned to detect a relative reduction of 60% in the percentage of PCV13 randomly assigned participants becoming experimental carriers compared with the saline randomly assigned individuals. With a total sample size of 200 individuals we calculated (using simulation) that we had 74% power to detect this difference. The code for the revised power calculation via simulation is available on GitHub.

The log-binomial model provides a direct estimation of the relative risk (RR) associated with the different predictor variables and, as such, has been recommended for use in randomised clinical trials.^20^ Specifically, the estimate of β_PCV13_, the model coefficient for the binary indicator variable for the study group, will estimate the average (for the study population) difference between study groups in the logarithm of the probability of experimental carriage (as defined by the primary study endpoint). This means that exp(β_PCV13_), the exponentiated model coefficient for the study group, will directly estimate the RR of experimental carriage associated with PCV13 vaccination. If this is less than 1, it means that the vaccine is protective and the vaccine efficacy can then be estimated as vaccine efficacy=1− exp(β_PCV13_). Log-binomial models can have difficulties with convergence, but we have used the logbin R package which mitigates these issues through the use of more stable expectation–maximisation-type algorithms.^21^

Categorical variables were compared by means of Fisher's exact test. A p value of less than 0·05 was considered significant; all p values are two tailed. The full statistical analysis plan, providing details of the planned analyses with all R code, is available from GitHub. The trial is registered with the Pan African Clinical Trials Registry, PACTR202008503507113.

### Role of the funding source

The funder of the study had no role in study design, data collection, data analysis, data interpretation, or writing of the report.

## Results

**Figure 1:**
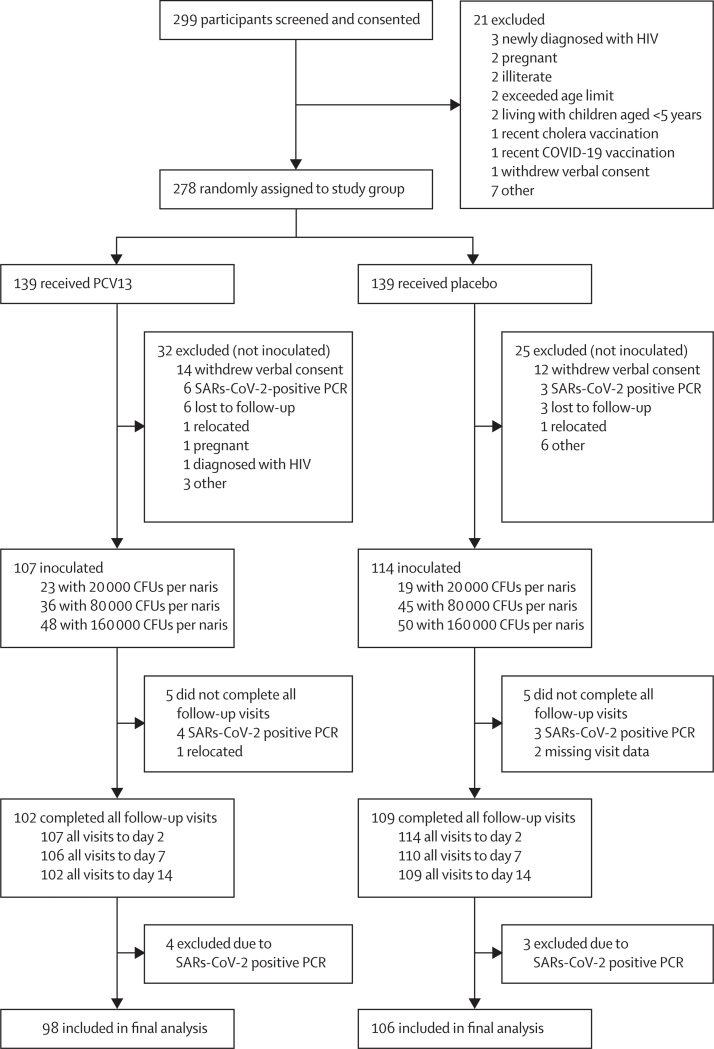
Trial profile Some participants were excluded after a delay of >10 weeks between vaccination and inoculation per protocol.^17^ Delays of >10 weeks occurred primarily due to a national wave of COVID-19 infection in Malawi leading to a pause of study activities between June and September, 2021. CFU=colony-forming unit. PCV13=13-valent pneumococcal conjugate vaccine.

Participant recruitment was continuous between April 27, 2021, and Aug 9, 2022, with a brief pause (June 8, 2021–Sept 13, 2021) owing to a national COVID-19 infection wave in Malawi requiring staff to assist with hospital emergency cover.^22^, ^23^ The screening, vaccination, and exclusion of participants in the study are shown in figure 1. Participants with SARS-CoV-2 infection were unable to attend the research clinic for scheduled visits and this resulted in missing data in seven participants following inoculation (another seven participants also tested positive for SARS-CoV-2 but did complete all study visits; in total, 14 participants infected with SARS-CoV-2 were removed from analysis). Groups randomly assigned to receive either PCV13 or placebo were broadly similar in terms of age, BMI, and smoking history, and the majority were men; participant characteristics are presented in table 1.

**Table 1:** Participant characteristics by randomisation group

		**Placebo**	**PCV13**	**Overall**
Study characteristics				
	Participants	114 (52%)	107 (48%)	221 (100%)
	SARS-CoV-2 infection	6 (5%)	8 (7%)	14 (6%)
	Completed follow-up visits and included in final analysis*	106 (93%)	98 (92%)	204 (92%)
Sex				
	Male	72 (68%)	75 (77%)	147 (72%)
	Female	34 (32%)	23 (23%)	57 (28%)
Age, years		24·6 (22·8–28·5)	25·5 (23·1–28·0)	25·3 (22·9–28·5)
BMI, kg/m^2^		22·0 (20·3–23·7)	21·5 (20·2–24·5)	21·8 (20·2–23·9)
Smoking status				
	Never smoker	104 (98%)	95 (97%)	199 (98%)
	Past smoker	2 (2%)	2 (2%)	4 (2%)
	Current smoker	0	1 (1%)	1 (<1%)

Data are n (%) or median (IQR). PCV13=13-valent pneumococcal conjugate vaccine.

* The totals from this row are used as the column-wide denominators to calculate percentages in rows further down in the table.

All inoculation doses were within the target range (doubling or halving of target dose). The mean inoculation doses at each target dose were 19 056 CFUs per 100 μL (SD 2808 CFUs per 100 μL) for the 20 000 CFUs per 100 μL target; 75 185 CFUs per 100 μL (13 333 per 100 μL) for the 80 000 CFUs per 100 μL target; and 155 493 per 100 μL (32 738 per 100 μL) for 160 000 CFUs per 100 μL target (appendix p 4).

There were no vaccination or inoculation related serious adverse events, but one serious adverse event was recorded in a participant with SARS-CoV-2 infection who required medical attention. Adverse events were mild and unrelated to pneumococcal vaccination, inoculation, or carriage. All adverse events are reported in the appendix (pp 1–2)—nasal washing resulted in nasal stuffiness in 18 (8%) of 221 participants and there were a wide variety of mild, non-specific symptoms reported in direct questioning in the weeks following inoculation.

Experimentally induced carriage of *S pneumoniae* 6B was established by culture of nasal wash in two (5%) of 40 volunteers inoculated at 20 000 CFUs per naris, 18 (24%) of 74 inoculated at 80 000 CFUs per naris, and 20 (22%) of 90 inoculated at 160 000 CFUs per naris. Data for the percentage of volunteers found to have *S pneumoniae* 6B carriage by culture in nasal wash at each timepoint are shown in table 2.

**Table 2:** Participants with confirmed *Streptococcus pneumoniae* serotype 6B (strain BHN418) nasal carriage by microbiological culture from nasal wash

	**Placebo**		**PCV13**	
	*S pneumoniae* serotype 6B carriers	Proportion (95% CI)	*S pneumoniae* serotype 6B carriers	Proportion (95% CI)
**Day 2, CFU per naris**				
20 000	2/19	10·5% (1·3–33·1)	0/21	0·0% (0·0–16·1)
80 000	11/41	26·8% (14·2–42·9)	4/33	12·1% (3·4–28·2)
160 000	9/46	19·6% (9·4–33·9)	4/44	9·1% (2·5–21·7)
**Day 7, CFU per naris**				
20 000	1/19	5·3% (0·1–26.0)	0/21	0·0% (0·0–16·1)
80 000	8/41	19·5% (8·8–34·9)	3/33	9·1% (1·9–24·3)
160 000	9/46	19·6% (9·4–33·9)	3/44	6·8% (1·4–18·7)
**Day 14, CFU per naris**				
20 000	1/19	5·3% (0·1–26·0)	0/21	0·0% (0·0–16·1)
80 000	8/41	19·5% (8·8–34·9)	1/33	3·0% (0·1–15·8)
160 000	10/46	21·7% (10·9–36·4)	3/44	6·8% (1·4–18·7)
All visits,*CFU per naris				
20 000	2/19	10·5% (1·3–33·1)	0/21	0·0% (0·0–16·1)
80 000	12/41	29·3% (16·1–45·5)	6/33	18·2% (7·0–35·5)
160 000	16/46	34·8% (21·4–50·2)	4/44	9·1% (2·5–21·7)

Data are n/N, proportion (%), or 95% CI. Nasal wash samples were taken 2 days (visit E), 7 days (visit F) and 14 days (visit G) after bacterial inoculation. Differential carriage rates are categorised by inoculation dose and by randomisation allocation (intramuscular saline *vs* PCV13 28 days before bacterial inoculation). CFU=colony forming unit. PCV13=13-valent pneumococcal conjugate vaccine.

* This row aggregates carriage data for individual participants across all three visits.

The experimental carriage rate at each dose was lower in unvaccinated participants than expected. We modelled the dose–response curve (appendix p 5) and showed a plateau function that suggested that further increasing the inoculation dose would not reach the anticipated 60% carriage rate.

**Figure 2:**
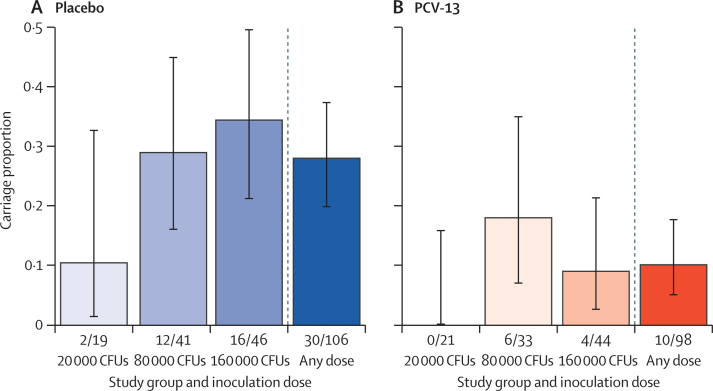
*Streptococcus pneumoniae* serotype 6B (BHN418) carriage by study group Participants randomly assigned to intramuscular 0·9% saline *vs* PCV13 and inoculation dose. The figure represents carriage proportions by inoculation dose, comparing saline group (A) with PCV13 (B). Number of carriers of total participants are indicated below each bar. Log-binomial regression p value for PCV13 vaccination coefficient, p=0·0033. Fisher's exact test (overall carriage), p=0·0013. Fisher's exact test p value: at doses 20 000 CFU per naris, p=0·22, 80 000 CFUs per naris, p=0·29, and 160 000 CFUs per naris, p=0·005. CFU=colony forming unit. PCV13=13-valent pneumococcal conjugate vaccine.

The rate of experimental *S pneumoniae* 6B carriage is compared by vaccine and placebo groups in figure 2. At every dose, there were fewer participants with experimental *S pneumoniae* 6B carriage in the vaccine group compared with placebo (figure 2). At 20 000 CFUs per naris, none of the vaccine group and two of the placebo group had *S pneumoniae* 6B experimental carriage. At 80 000 CFUs per naris there were six carriers in the vaccine group and 12 in the placebo group whereas in the 160 000 CFUs per naris, there were four carriers in the vaccine group and 16 in the placebo group. The log-binomial model adjusting for inoculation dose group showed a 62·4% protection of vaccine compared with placebo (carriage RR 0·38, 95% CI 0·20–0·72, p=0·0033). Our data do not show evidence for a difference in *S pneumoniae* 6B carriage risk between the 80 000 CFUs per naris and 160 000 CFUs per naris dose, although the log-binomial model did identify a significant reduction in carriage risk for the 20 000 CFUs per naris dose compared with the 160 000 CFUs per naris dose (RR 0·231, 95% CI 0·06–0·92, p=0·038; table 3).

**Table 3:** Summary of the log-binomial regression model fit

	Estimate*	**SE**	**Z statistic**	**p value**
Intercept	0·327	0·201	−5·573	<0·0001
PCV13	0·376	0·333	−2·936	0·0033
20 000 CFUs per naris	0·231	0·706	−2·077	0·038
80 000 CFUs per naris	1·000	0·276	0·000	1·000

The baseline carriage risk for a saline-randomised participant inoculated at the CFU 160 000 dose is 33% (95% CI 22·0–48·4). There is a significant effect of PCV13 vaccine on the probability of serotype 6B carriage (risk ratio 0·38, 95% CI 0·20–0·72, p=0·0033). CFU=colony forming unit. PCV13=13-valent pneumococcal conjugate vaccine.

* Shows the baseline risk on the Intercept row and the relative risk associated with each of the exposures on the subsequent rows.

**Figure 3:**
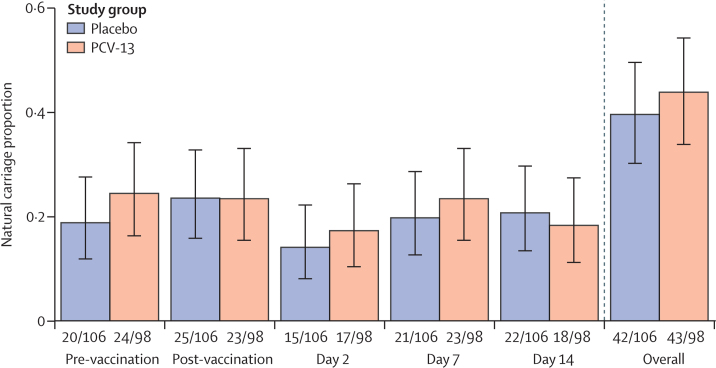
Natural *Streptococcus pneumoniae* nasal carriage by study visit and study group Participants randomly assigned to intramuscular 0·9% saline *vs* PCV13. The figure shows natural carriage proportions per study group at the different study visits. The right-most pair of bars show overall carriage, defined as carriage at any study visit. No *S pneumoniae* serotype 6B (BHN418) natural carriage episodes were observed before experimental challenge (day 0). PCV13=13-valent pneumococcal conjugate vaccine.

As expected from community prevalence data,^3^ there were high rates of non-experimental pneumococcal carriage in the study groups. A comparison of natural carriage rates (non-*S pneumoniae* 6B) by vaccine group at each timepoint (pre-vaccination, post-vaccination, day 2, day 7, and day 14 post-inoculation) is shown in figure 3. Data on vaccine-type and non-vaccine-type natural carriage are shown in the appendix (p 6). Natural carriage rates of approximately 20% were found at each timepoint in both PCV13 vaccinated and placebo groups, with 85 (42%) of 204 participants having natural carriage from the point of enrolment to study exit.

The primary analysis of vaccine effect on experimental pneumococcal (*S pneumoniae* 6B) carriage was repeated for the group with no natural carriage pre-inoculation and separately for the group with natural pneumococcal carriage at the pre-inoculation visit. These analyses showed a greater protective efficacy of vaccine in the group without natural carriage (69·1%) compared with the group with established natural carriage at the time of inoculation (51·7%; table 4).

**Table 4:** Tabulated counts and percentages of experimental *Streptococcus pneumoniae* serotype 6B (BHN418) carriers stratified by natural carriage and randomisation group

	S pneumoniae **6B carriers**	**Proportion of *S pneumoniae* 6B carriers (95% CI)**	**Vaccine efficacy**
**No natural carriage**			
Placebo	21/81	25·9% (16·8–36·9)	NA
PCV13	6/75	8·0% (3·0–16·6)	69·1%
**Natural carriage**			
Placebo	9/25	36·0% (18·0–57·5)	NA
PCV13	4/23	17·4% (5·0–38·8)	51·7%

Natural carriage is defined here as being a carrier of a non-6B strain at the pre-inoculation visit. PCV13=13-valent pneumococcal conjugate vaccine. NA=not applicable.

## Discussion

We have shown in a double-blind, randomised controlled trial, which made use of a human infection study that PCV13 has a protective effect against experimental pneumococcal carriage in healthy young Malawian adults. This is an important result because of the disappointingly high rates of vaccine-type pneumococcal carriage in our context, following national vaccine roll-out. The study offers both reassurance in showing a vaccine effect, and an explanation for the observed persistent carriage in showing a reduced vaccine effect associated with natural carriage at the time of experimental inoculation. It could be that high rates of natural carriage lead to reduced vaccine effectiveness. This is also a landmark study in that it is, to our knowledge, the first vaccine trial that makes use of a bacterial human infection study in Africa.

There are similarities and differences between our study and the experimental human carriage randomised controlled trial of PCV13 published in Liverpool.^14^ The clear protective effect of vaccine against carriage is seen in both studies, but is less in Malawi (62%, 95% CI 28–80) than in Liverpool (78%, 48–91). The CIs of these estimates overlap, but the lower value in Malawi is consistent with the observed persistent vaccine-type carriage (18%) in vaccinated children^3^ compared with no persistent vaccine-type carriage in the UK.^24^ The human infection studies were both carried out in healthy young adults and resulted in no vaccine or inoculation related adverse events.

The first major difference in the human infection studies in Malawi and Liverpool was that more Malawian participants were male compared with the Liverpool study (72% male in Malawi *vs* 40% in Liverpool, UK).^14^ The difference in sex between the two studies is of interest as it reflects enthusiasm for human infection studies and participation in research in general in both settings. It has been shown before that male sex is associated with lower experimental carriage rates, but a mechanism has not been proposed as that study, like this one, excluded women caring for young children.^25^

The second major difference between the UK and Malawi was the difference in natural carriage. In Malawi, more participants had natural pneumococcal carriage during the study than in Liverpool. 42% of young Malawian adults had an episode of natural carriage during the 2 months of the study compared with only 6% in Liverpool^14^—and those with natural carriage at the point of inoculation had a reduced experimental carriage rate compared with those without pneumococcal carriage at that moment. This result is very likely to be relevant to children who have high rates of carriage (>80%) in our context. We do not have an explanation for the reduced vaccine effect in participants with pre-existing natural carriage but there might be an immunological mechanism preventing optimal antigen presentation of vaccine in the presence of an ongoing carriage. Regulatory T-cell responses are known to be crucial in persistent carriage.^26^

In the context of the experimental model, these observations suggest that the ecological niche into which the experimental inoculum is pipetted is a determinant of success or otherwise in establishing experimental carriage. There are other data to support this hypothesis. We have described nasopharyngeal microbiomes in which high diversity was associated with experimental carriage, and lower diversity with less carriage.^27^ More recently, experimental data from a murine model indicate a hierarchy of success dependent on pneumococcal serotype—the importance of this concept can only be tested by further work in our model with additional serotypes.^28^ It might be that pneumococcal colonisation alters the human nasopharyngeal microbiome to make acquisition of further pneumococcal serotypes more efficient. Further, we have described an association between asymptomatic viral colonisation and the efficiency of experimental colonisation.^29^, ^30^ Studies to establish the viral colonisation of the Malawian participants in this study are in progress. If natural carriage determines experimental carriage, then this factor, and the effect of sex, must be included in vaccine evaluation studies in order not to underestimate the protective efficacy of vaccine.

A limitation of our study is the low rate of experimental carriage that was achieved. We increased the inoculation dose to twice that used in Liverpool. Further, we modelled the dose–response function (appendix p 5) and showed that a plateau effect was evident. This curve suggests that little further carriage would have been achieved by the use of higher inoculation doses. In fact, murine data^31^ suggest that very high doses might result in inflammation and resulting lower rates of experimental carriage.

The primary purpose of this study was to establish, by means of a human infection study, whether PCV13 was protective against carriage in Malawian adults. This result was clear and showed that vaccination does protect against carriage. This result has been shown to be similar in both Liverpool, UK and Blantyre, Malawi, suggesting that the result is generalisable, albeit with consideration of sex and natural carriage incidence. PCV13 boosts circulating anticapsular IgG,^32^ but also affects humoral and cellular responses in other compartments. The human infection study method allows sampling of serum and nasal fluid for humoral defenses, and nasal mucosa and blood for cellular defense.^29^ These studies might suggest means by which the vaccine efficacy of PCV13 to prevent vaccine type carriage might be improved.

**This online publication has been corrected. The corrected version first appeared at thelancet.com/microbe on October 4, 2023**

## Data sharing

An anonymised study dataset can be shared within Malawi in line with local data sharing policy. Requests for data sharing outside Malawi can be presented to the Data Research Support Unit, Malawi–Liverpool–Wellcome Research Programme (https://www.mlw.mw/departments/statistical-support-unit/). A full dataset combining Liverpool and Malawi data is in preparation and analyses from this dataset are planned.

## Declaration of interests

DF declares grant funding from Pfizer to her institution for separate projects and consulting fees from Pfizer, MSD, and Sanofi. All other authors declare no competing interests.
