# Correction to *Lancet Microbe* 2023; 4: e683–91

**DOI:** 10.1016/S2666-5247(23)00322-1

**Published:** 2023-11

*Dula D, Morton B, Chikaonda T, et al. Effect of 13-valent pneumococcal conjugate vaccine on experimental carriage of* Streptococcus pneumoniae *serotype 6B in Blantyre, Malawi: a randomised controlled trial and controlled human infection study.* Lancet Microbe *2023; **4:** e683–91*—In this article in the Research in context, Methods, and Results sections, the unit of volume is incorrect, it should be “100 μL”. Likewise, in the Procedures section, the final sentence in the second paragraph should read “800 μL skim milk, tryptone, glucose, and glycerol was added to the remaining nasal wash pellet and divided into three vials (300 μL into…” These corrections have been made as of Oct 4, 2023.

