# Supplementary material for: Effect of 13-valent pneumococcal conjugate vaccine on experimental carriage of Streptococcus pneumoniae serotype 6B in Blantyre, Malawi: a randomised controlled trial and controlled human infection study
Source: Lancet Microbe. 2023 Sep;4(9):e683–91. doi: 10.1016/S2666-5247(23)00178-7 (PMC10469263; doi:10.1016/S2666-5247(23)00178-7)
Supplement: Supplementary appendix [file mmc1.pdf]

THE LANCET
Microbe

Supplementary appendix

This appendix formed part of the original submission and has been peer reviewed. We post it as supplied by the authors.

Supplement to: Dula D, Morton B, Chikaonda T, et al. Effect of 13-valent pneumococcal conjugate vaccine on experimental carriage of *Streptococcus pneumoniae* serotype 6B in Blantyre, Malawi: a randomised controlled trial and controlled human infection study. *Lancet Microbe* 2023; **4:** e683–91.

Supplementary Material

Contents

Supplementary Table S1. Adverse Events per inoculation dose and severity. * indicated that an individual participant had more than 1 AE, CHIMB2561* was identified as a SAE (required medical attention following SARS-CoV-2 infection) 1
Supplementary Table S2. Adverse events by vaccination arm and inoculation dose (aggregated) 3
Supplementary Figure S1: Inoculation doses of *Streptococcus pneumoniae* serotype 6B (SPN6B) administered to participants within the trial 4
Supplementary Figure S2: Probability of experimental *Streptococcus pneumoniae* serotype 6B (SPN6B) experimental carriage dose-response curve estimated using SPN6B inoculation doses from study participants randomised to intramuscular saline control injection 5
Supplementary Figure S3: Natural *Streptococcus pneumoniae* nasal carriage by study visit and study arm (participants randomised to intramuscular 0.9% saline vs PCV-13 vaccination), stratified by vaccine type (PCV13) and non-vaccine type *Streptococcus pneumoniae* 6
Reflexivity statement 7
Reporting checklist for randomised trial 10
Consortium members 14

Supplementary Table S1. Adverse Events per inoculation dose and severity. *indicated that an individual participant had more than 1 AE, CHIMB2561* was identified as a SAE (required medical attention following SARS-CoV-2 infection).

PID	Study arm	Dose	Pre-inoculation visit date	Symptoms	Severity	Relation to inoculation or vaccination	Time since pre-inoculation visit (days)
**Included in the final analysis**							
CHIMB1035	PCV-13	20,000 CFU	19-May-21	rash	mild	unlikely	20
CHIMB1068		20,000 CFU	25-May-21	headache	mild	unlikely	13
CHIMB1118		20,000 CFU	26-May-21	coryzal, cough	mild	unlikely	20
CHIMB1134		20,000 CFU	02-Jun-21	coryzal	mild	unlikely	19
CHIMB1266		20,000 CFU	10-Jun-21	sore throat, headache	mild	unlikely	12
CHIMB1910		80,000 CFU	31-Jan-22	sore throat, cough, headache	mild	unlikely	9
CHIMB1985		80,000 CFU	31-Jan-22	sore throat	mild	unlikely	14
CHIMB2256*		80,000 CFU	15-Mar-22	fever, headache	mild	unlikely	9
CHIMB2256*		80,000 CFU	15-Mar-22	sore throat, cough	mild	unlikely	14
CHIMB2355		80,000 CFU	22-Mar-22	cough	mild	unlikely	14
CHIMB2595		160,000 CFU	10-May-22	coryzal	mild	unlikely	14
CHIMB3155		160,000 CFU	21-Jun-22	coryzal, fever, headache, abdominal pains	mild	unlikely	9
CHIMB1183*	Saline	20,000 CFU	09-Jun-21	coryzal	mild	unlikely	7
CHIMB1183*		20,000 CFU	09-Jun-21	coryzal	mild	unlikely	19
CHIMB1191		20,000 CFU	09-Jun-21	coryzal	mild	unlikely	19
CHIMB1233		20,000 CFU	10-Jun-21	body pains	mild	unlikely	7
CHIMB1282		80,000 CFU	25-Oct-21	diarrhea	mild	unlikely	9
CHIMB1415		80,000 CFU	26-Oct-21	toung lession	mild	unlikely	14
CHIMB1530		80,000 CFU	26-Oct-21	coryzal, earache, fever	mild	unlikely	9
CHIMB1696		80,000 CFU	22-Nov-21	sore throat	mild	unlikely	9
CHIMB1753		80,000 CFU	11-Jan-22	sore throat, coryzal, headache	mild	unlikely	9

CHIMB1878		80,000 CFU	31-Jan-22	sore throat, coryzal, headache	mild	unlikely	9
CHIMB1977		80,000 CFU	31-Jan-22	headache	mild	unlikely	14
CHIMB2116		80,000 CFU	28-Feb-22	coryzal	mild	unlikely	21
CHIMB2249		80,000 CFU	15-Mar-22	cough	mild	unlikely	14
CHIMB2413		80,000 CFU	28-Mar-22	coryzal, headache	mild	unlikely	23
CHIMB2470		80,000 CFU	05-Apr-22	coryzal, headache	mild	unlikely	14
CHIMB2769		160,000 CFU	30-May-22	sore throat	mild	unlikely	14
CHIMB2777		160,000 CFU	23-May-22	cough	mild	unlikely	14
CHIMB2801		160,000 CFU	30-May-22	coryzal	mild	unlikely	7
CHIMB3015		160,000 CFU	21-Jun-22	headache	mild	unlikely	9
CHIMB3080		160,000 CFU	20-Jun-22	headache	mild	unlikely	9
CHIMB3106		160,000 CFU	14-Jun-22	sore throat	mild	unlikely	14
CHIMB3130*		160,000 CFU	21-Jun-22	coryzal	mild	unlikely	0
CHIMB3130*		160,000 CFU	21-Jun-22	Joint pain and restlessness	mild	unlikely	9
CHIMB3312		160,000 CFU	28-Jun-22	coryzal, cough	mild	unlikely	14
CHIMB3411		160,000 CFU	04-Jul-22	coryzal, cough	mild	unlikely	9
**Not included in the final analysis**							
CHIMB1522	PCV-13	Not inoculated	28-Sep-21	coryzal	mild	unlikely	0
CHIMB2561*		160,000 CFU	10-May-22	cough	mild	unlikely	9
CHIMB2561*		160,000 CFU	10-May-22	fever, headache	severe	unlikely	22
CHIMB1449	Saline	80,000 CFU	16-Nov-21	coryzal	mild	unlikely	9

Supplementary Table S2. Adverse events by vaccination arm and inoculation dose (aggregated).

Study arm	Dose	Total AEs	Total patients with AEs
**Included in the final analysis**			
PCV-13	20,000 CFU	6	5
	80,000 CFU	9	4
	160,000 CFU	5	2
	**Total**	**20**	**11**
Saline	20,000 CFU	4	3
	80,000 CFU	19	11
	160,000 CFU	12	9
	**Total**	**35**	23
**Not included in the final analysis**			
PCV-13	not inoculated	1	1
	160,000 CFU	3	1
Saline	80,000 CFU	1	1

Supplementary Figure S1: Inoculation doses of *Streptococcus pneumoniae* serotype 6B (SPN6B) administered to participants within the trial. Doses determined by classical microbiological culture from inoculum suspension. Dashed lines indicate per protocol tolerable ranges. Dots indicate individual data points whilst boxes and whiskers summarise distributions for each dose group (20,000, 80,000 and 160,000 CFUs respectively). CFU: colony forming unit.

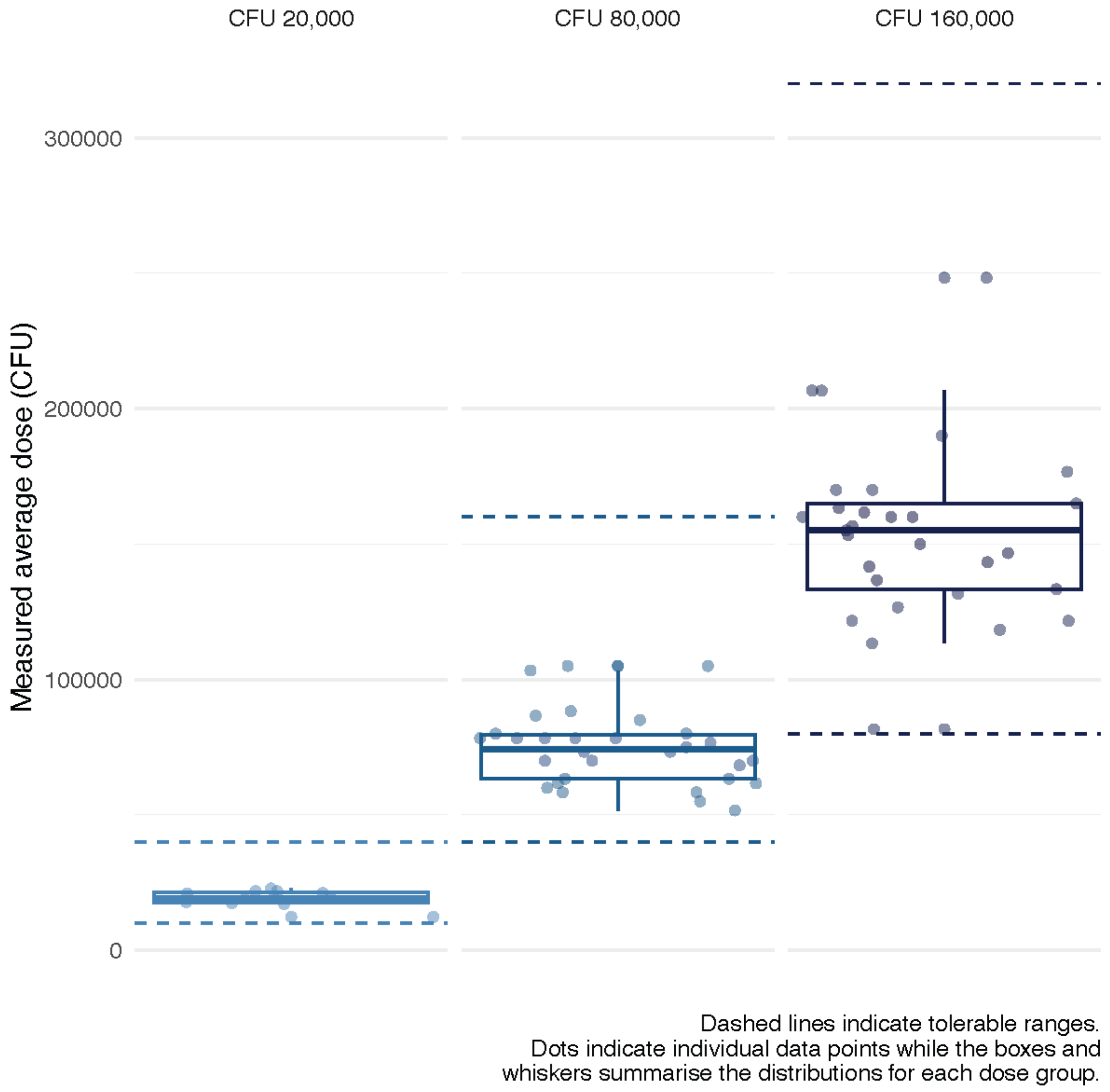

Supplementary Figure S2: Probability of experimental *Streptococcus pneumoniae* serotype 6B (SPN6B) experimental carriage dose-response curve estimated using SPN6B inoculation doses from study participants randomised to intramuscular saline control injection. A 2−parameter log−logistic model was used for the dose−response curve estimation. Grey dots show the individual carriage data as a function of actual dose delivered. Black dots with error bars show the estimated proportion of carriage by target dose and the associated 95% confidence intervals. Dashed blue lines and band indicate that the ED50 threshold dose was 326,311 CFU with the 95% CI (149,622, 23,121,362). CFU: colony forming unit.

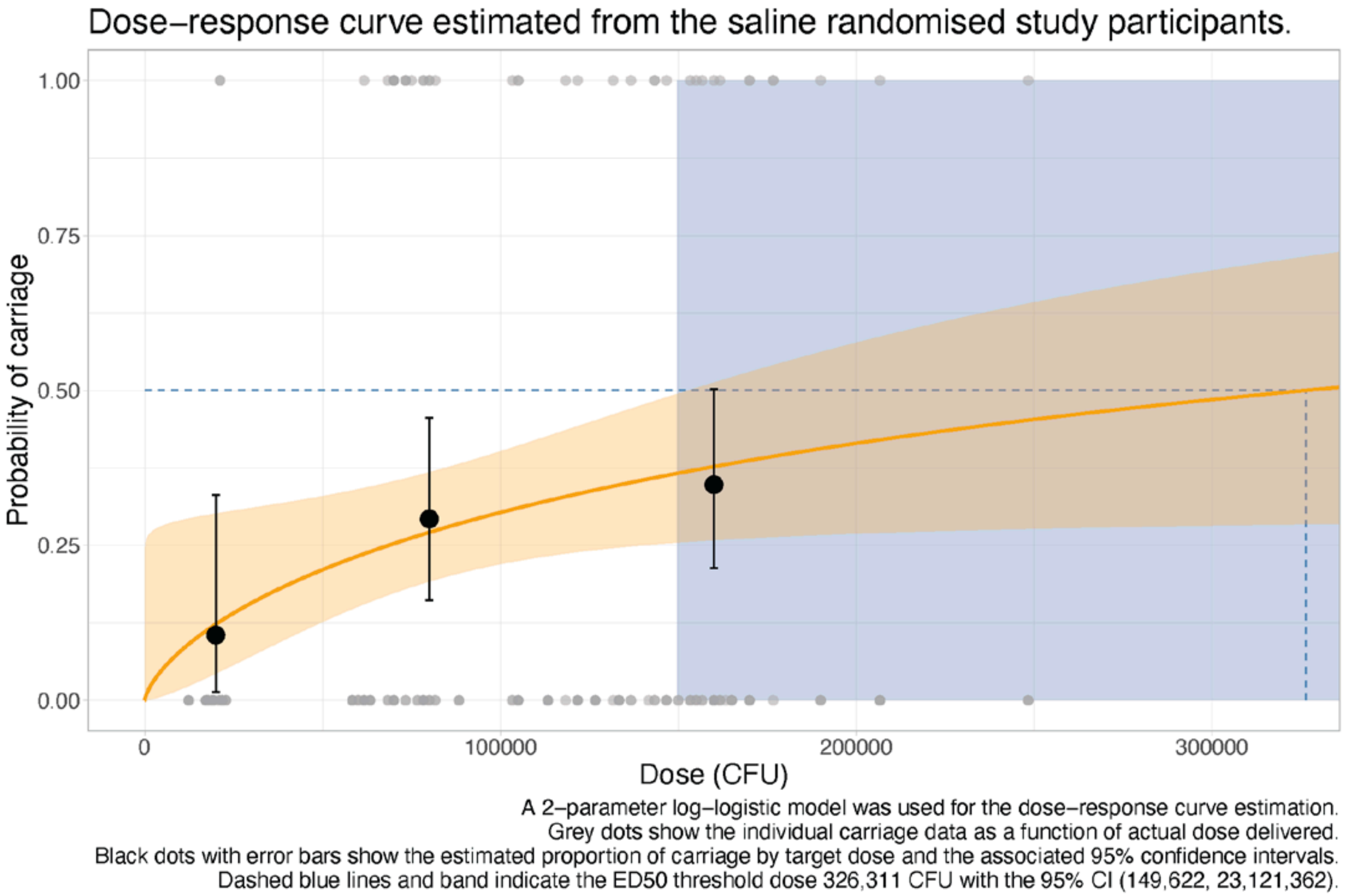

Supplementary Figure S3: Natural *Streptococcus pneumoniae* nasal carriage by study visit and study arm (participants randomised to intramuscular 0.9% saline vs PCV-13 vaccination), stratified by vaccine type (PCV13) and non-vaccine type *Streptococcus pneumoniae*. Figure demonstrates natural carriage proportions per study arm at the different study visits. The right-most pair of bars show overall carriage, defined as carriage at any study visit. No SPN6B natural carriage episodes were observed prior to experimental challenge (Day 0). PCV-13: Pneumococcal conjugate vaccine-13.

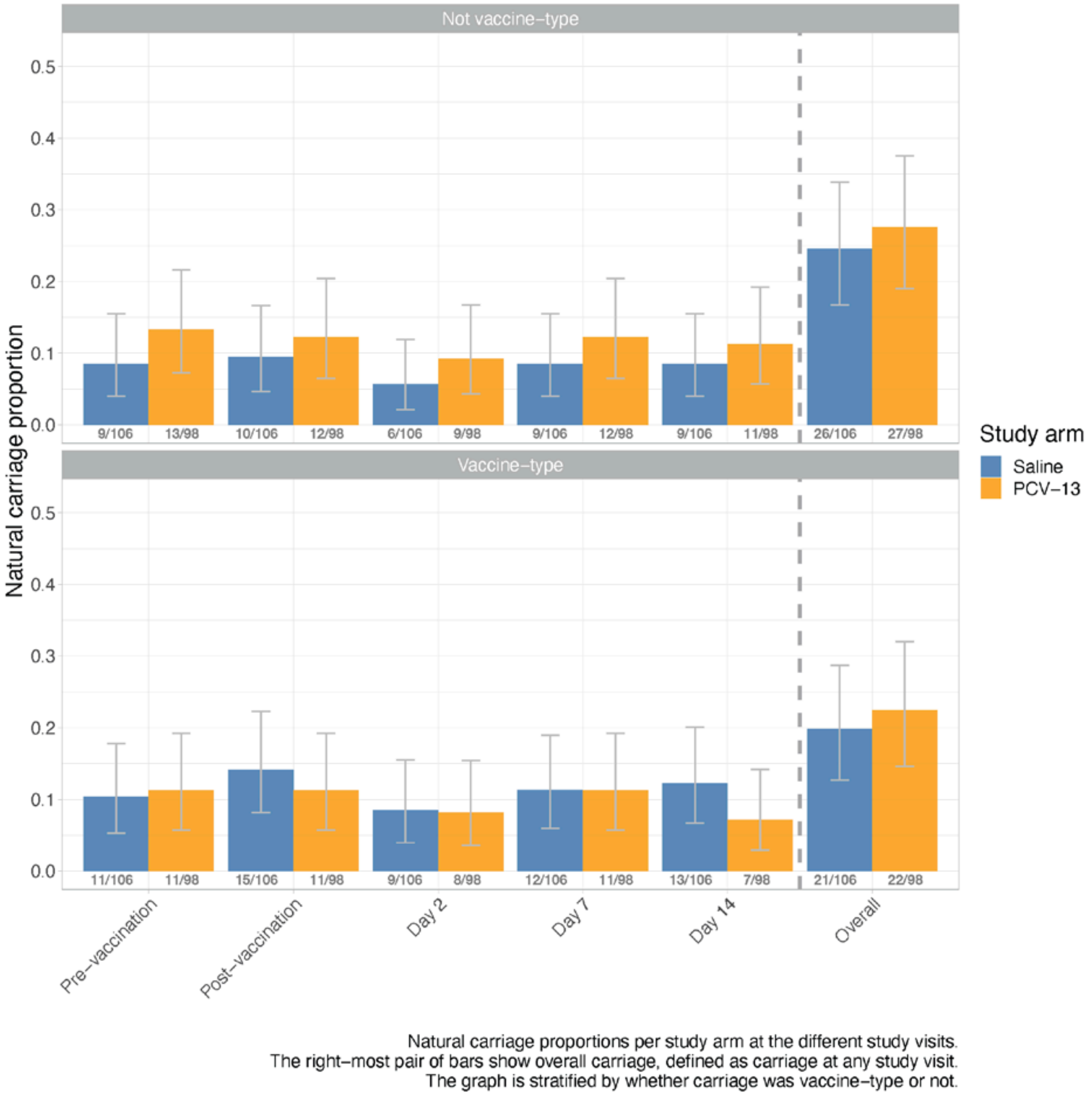

Reflexivity Statement

Randomised controlled trial of pneumococcal conjugate vaccine shows protection against experimental pneumococcal carriage in first human infection study in Africa

**Study conceptualization**	**1. How does this study address local research and policy priorities?**<br>Invasive pneumococcal disease is a major cause of infant mortality in Malawi. Since the introduction of PCV13 in 2011, there have been multiple studies demonstrating that the vaccine, whilst protecting individuals against disease is not promoting expected herd immunity effects (observed in HICs). As development increases and countries transition from GAVI funding, governments need to priortise health funding. PCV13 remains an expensive vaccine and local data to inform research and policy is required.
	**2. How were local researchers involved in study design?**<br>The MARVELS programme leadership includes the study chief investigator (Gordon); clinical leads (Dula/Morton); microbiology lead (Chikaonda); and immunology lead (Jambo). The multidisciplinary study protocol for this trial was primarily drafted by Morton (Dula took on clinical leadership in July 2021), Chikaonda and Jambo. Dula, Chikaonda and Jambo are all senior researchers from Malawi.
**Research management**	**3. How has funding been used to support the local research team(s)?**<br>The MARVELS programme has supported local researchers to develop skills and expertise including both formal and informal training opportunities. These include clinical, microbiological and immunological junior research staff. For example, Nsomba and Chirwa are both enrolled in Master's degree programmes and Sichone is now registered for a PhD.
**Data acquisition and analysis**	**4. How are research staff who conducted data collection acknowledged?**<br>All research staff who conducted data collection are included in the authorship of the manuscript.
	**5. How have members of the research partnership been provided with access to study data?**<br>Study data is archived at Malawi Liverpool Wellcome Trust. All researchers have access to study data which is discussed at multidisciplinary weekly team meetings.
	**6. How were data used to develop analytical skills within the partnership?**

	Dingase Dula is an early career researcher who has worked closely with our trial statistician (Henrion) to develop analytical skills. Further, Henrion offers a “R for beginners” course for all researchers involved within the programme. Jambo is training lead for the Malawi-Liverpool Wellcome Trust and offers immunological analytical training to junior researchers within this programme.
**Data interpretation**	**7. How have research partners collaborated in interpreting study data?**<br>Data is discussed and interpreted by the team at weekly team meetings. For example, junior members of the microbiology team presented both classical and molecular data on nasal pneumococcal carriage and worked with the study team to interpret and contextualise these findings
**Drafting and revising for intellectual content**	**8. How were research partners supported to develop writing skills?**<br>We have held writing retreats during the course of developing this manuscript, offering senior feedback and coaching to develop writing skills for junior researchers.
	**9. How will research products be shared to address local needs?**<br>This manuscript will be shared with local partners in the Ministry of Health, participants and health care workers in local hospitals. We will make presentations at local research dissemination conferences and at routine meetings at the hospital where the study was conducted, and within the research institution (MLW)
**Authorship**	**10. How is the leadership, contribution and ownership of this work by LMIC researchers recognised within the authorship?**<br>Please refer to the section on “Authors’ contribution” in the manuscript. Dingase Dula is a female early career researcher who has led as joint first author on this manuscript.
	**11. How have early career researchers across the partnership been included within the authorship team?**<br>We have included multiple early career researchers within the authorship of this manuscript including Dula, Chirwa, Nsomba, Nkhoma, Ngoliwa, Sichone, Galafa, Tembo, Chaponda, Kamng’ona, Kudowa, Howard, Mkandawire and Chimgoneko.
	**12. How has gender balance been addressed within the authorship?**

	Eleven out of 27 (40.7%) members of the named authorship team are female
**Training**	**13. How has the project contributed to training of LMIC researchers?**<br>Multiple researchers have been trained as part of this project. We have transferred established laboratory SOPs from Liverpool to Malawi and all assays were conducted in our laboratories at MLW. This includes training in microbiological and immunological procedures conforming to robust standards, inspected by an external CRO. Our clinical team have been trained in robust safety procedures for controlled human infection model research and as critical care nurses/doctors delivering patient care at the local hospital for the clinical component of their job plans. A number of our team have enrolled in Masters programmes supported by this programme including Nsomba and Chirwa.
**Infrastructure**	**14. How has the project contributed to improvements in local infrastructure?**<br>This project has directly contributed to improvements in both the research and clinical restructureure locally. As per Q13, our team support clinical care at the local hospital as part of a refurbished medical high dependency unit. The programme has also supported refuribishment of research laboratories including a dedicated space to safely prepare and culture experimental pathogen challenge agents
**Governance**	**15. What safeguarding procedures were used to protect local study participants and researchers?**<br>The local ethics body and LSTM research ethics committee reviewed and approved the study protocol ensuring that both participants and researchers are protected throughout the study. We observed robust safeguarding procedures per sponsor requirements (https://www.lstmed.ac.uk/safeguarding/safeguarding-information-point) during this study.

Reporting checklist for randomised trial.

Based on the CONSORT guidelines.

Instructions to authors

Complete this checklist by entering the page numbers from your manuscript where readers will find each of the items listed below.

Your article may not currently address all the items on the checklist. Please modify your text to include the missing information. If you are certain that an item does not apply, please write "n/a" and provide a short explanation.

Upload your completed checklist as an extra file when you submit to a journal.

In your methods section, say that you used the CONSORTreporting guidelines, and cite them as:

Schulz KF, Altman DG, Moher D, for the CONSORT Group. CONSORT 2010 Statement: updated guidelines for reporting parallel group randomised trials

		Reporting Item	Page Number
**Title and Abstract**			
Title	#1a	Identification as a randomized trial in the title.	1
Abstract	#1b	Structured summary of trial design, methods, results, and conclusions	2
**Introduction**			
Background and objectives	#2a	Scientific background and explanation of rationale	5
Background and objectives	#2b	Specific objectives or hypothesis	5-6
**Methods**			
Trial design	#3a	Description of trial design (such as parallel, factorial) including allocation ratio.	7
Trial design	#3b	Important changes to methods after trial commencement (such as eligibility criteria), with reasons	8

Participants	#4a	Eligibility criteria for participants	7
Participants	#4b	Settings and locations where the data were collected	7
Interventions	#5	The experimental and control interventions for each group with sufficient details to allow replication, including how and when they were actually administered	9-10
Outcomes	#6a	Completely defined prespecified primary and secondary outcome measures, including how and when they were assessed	10
Outcomes	#6b	Any changes to trial outcomes after the trial commenced, with reasons	10-11
Sample size	#7a	How sample size was determined.	7-8
Sample size	#7b	When applicable, explanation of any interim analyses and stopping guidelines	7-8
Randomization - Sequence generation	#8a	Method used to generate the random allocation sequence: page 8	
Randomization - Sequence generation	#8b	Type of randomization; details of any restriction (such as blocking and block size): page 8	
Randomization - Allocation concealment mechanism	#9	Mechanism used to implement the random allocation sequence (such as sequentially numbered containers), describing any steps taken to conceal the sequence until interventions were assigned	8
Randomization - Implementation	#10	Who generated the allocation sequence, who enrolled participants, and who assigned participants to interventions	8
Blinding	#11a	If done, who was blinded after assignment to interventions (for example, participants, care providers, those assessing outcomes) and how.	7-8

Blinding	#11b	If relevant, description of the similarity of interventions	NA
Statistical methods	#12a	Statistical methods used to compare groups for primary and secondary outcomes	10-11
Statistical methods	#12b	Methods for additional analyses, such as subgroup analyses and adjusted analyses	10-11
**Results**			
Participant flow diagram (strongly recommended)	#13a	For each group, the numbers of participants who were randomly assigned, received intended treatment, and were analysed for the primary outcome	Figure 1
Participant flow	#13b	For each group, losses and exclusions after randomization, together with reason	Figure 1
Recruitment	#14a	Dates defining the periods of recruitment and follow-up	12
Recruitment	#14b	Why the trial ended or was stopped	12
Baseline data	#15	A table showing baseline demographic and clinical characteristics for each group	Table 1
Numbers analysed	#16	For each group, number of participants (denominator) included in each analysis and whether the analysis was by original assigned groups	Table 2
Outcomes and estimation	#17a	For each primary and secondary outcome, results for each group, and the estimated effect size and its precision (such as 95% confidence interval)	Table 2<br>12-123
Outcomes and estimation	#17b	For binary outcomes, presentation of both absolute and relative effect sizes is recommended	Tables 2 & 3
Ancillary analyses	#18	Results of any other analyses performed, including subgroup analyses and adjusted	Table 4

		analyses, distinguishing pre-specified from exploratory	
Harms	#19	All important harms or unintended effects in each group (For specific guidance see CONSORT for harms)	Tables S1 & S2
**Discussion**			
Limitations	#20	Trial limitations, addressing sources of potential bias, imprecision, and, if relevant, multiplicity of analyses	15
Generalisability	#21	Generalisability (external validity, applicability) of the trial findings	14-15
Interpretation	#22	Interpretation consistent with results, balancing benefits and harms, and considering other relevant evidence	14-15
Registration	#23	Registration number and name of trial registry	2
**Other information**			
Interpretation	#22	Interpretation consistent with results, balancing benefits and harms, and considering other relevant evidence	14-15
Registration	#23	Registration number and name of trial registry	7
Protocol	#24	Where the full trial protocol can be accessed, if available	8
Funding	#25	Sources of funding and other support (such as supply of drugs), role of funders	2, 11, 16

None The CONSORT checklist is distributed under the terms of the Creative Commons Attribution License CC-BY. This checklist can be completed online using https://www.goodreports.org/, a tool made by the EQUATOR Network in collaboration with Penelope.ai

Consortium Members

First name and middle name	Surname
Mark	Alderson
Sandra	Antoine
Christopher, R	Bailey
Debbie	Bogaert
Jeremy	Brown
Sarah	Burr
Marien, L	De Jonge
Klara	Doherty
David	Goldblatt
Gabriela	Gomes
Joel	Gondwe
Kate	Gooding
Jonathon	Grigg
Tina	Harawa
Rob	Heyderman
Jason	Hinds
Angela	Hyder-Wright
Simon	Jochems
Blessings	Kapumba
Vella	Kaudzu
Robert	Kneller
Richard	Malley
Jane	Mallewa
Lucinda	Manda-Taylor
Edward	Mangani
Mphatso	Mayuni
Henry	Mwandumba
Percy	Mwenechanya
Mike	Parker
Andrew	Pollard
Modesta	Reuben
Jeffrey	Weiser
